# First person – Abigail Ama Koomson and Patrice Delaney

**DOI:** 10.1242/bio.060367

**Published:** 2024-03-06

## Abstract

First Person is a series of interviews with the first authors of a selection of papers published in Biology Open, helping researchers promote themselves alongside their papers. Abigail Ama Koomson and Patrice Delaney are co-first authors on ‘ Sustained effects of developmental exposure to inorganic arsenic on hepatic *gsto2* expression and mating success in zebrafish’, published in BiO. Abigail and Patrice conducted the research described in this article while Capstone student (Abigail) and PhD candidate (Patrice) in Dr Kirsten Sadler Edepli's lab at New York University Abu Dhabi. Abigail is now a PhD Student at Yale School of Medicine, New Haven, investigating the mechanisms underlying liver disease. Patrice is a Postdoctoral Research Fellow in the lab of Dr Wolfram Goessling at Harvard Medical School, Boston, focused on how aberrant exposure to anthropogenic contaminants, such as bisphenol A, interfere with hormone-regulated events in liver development.


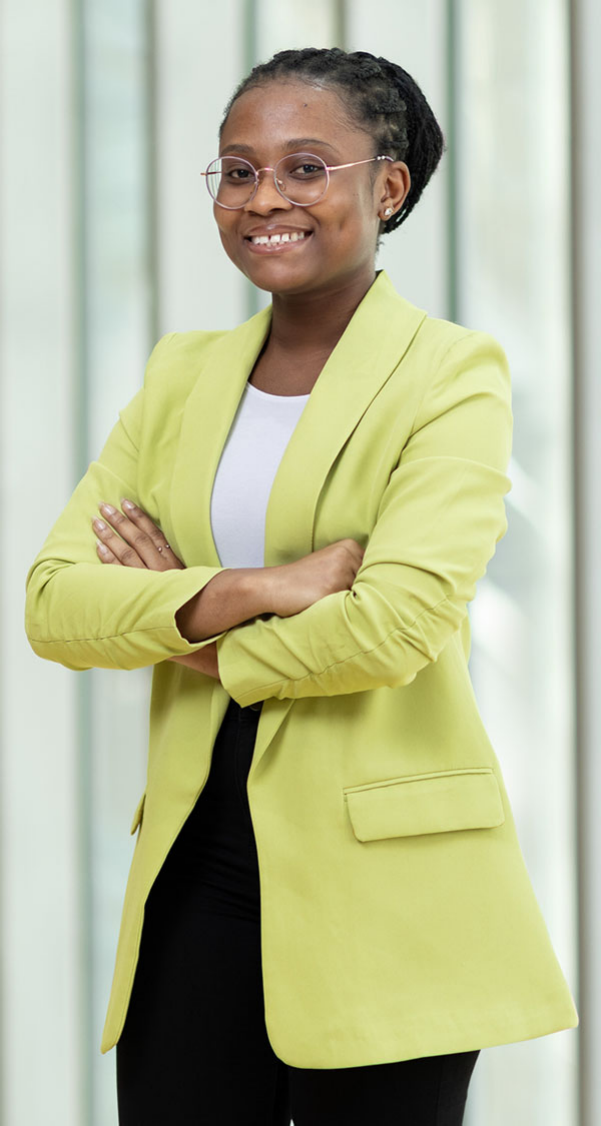


**Abigail Ama Koomson**

**Describe your scientific journey and your current research focus**

**Abigail:** My first introduction to research was as an undergraduate student in the lab of Dr Kirsten Sadler Edepli and there I worked on investigating the sustained effects of developmental exposure to inorganic arsenic on the liver. Currently, as a first-year PhD student at Yale, I have broad interests in the genetics of rare liver disease, exercise physiology and tumour metabolism.

**Patrice:** After completing my Bachelor of Arts from Sarah Lawrence College, I explored different careers by serving as a Science Educator at The Maritime Aquarium, an Administrative Assistant at Memorial Sloan Kettering Cancer Center, and as a Research Technician at New York University Abu Dhabi. During my role as a technician, I decided to further my education. This led me to pursue a Masters and PhD at New York University. I had the privilege of joining Dr Sadler's lab where I focused on the relationship between inorganic arsenic and liver disease. After defending my thesis, I joined Dr Wolfram Goessling's lab where I am currently focused on how aberrant exposure to anthropogenic contaminants, such as bisphenol A, interfere with hormone-regulated events in liver development and function.

**Who or what inspired you to become a scientist?**

**Abigail:** Prior to doing scientific research, I'd always thought of science as this neatly packaged knowledge in textbooks that I had to learn to pass my quizzes and exams. However, being surrounded by such a diverse group of talented scientists (female) in the Sadler lab and seeing them *do* science at the bench, in the fish room and at lab meetings helped me envision a place for myself in science. Again, recognizing that as a scientist, I had the unique opportunity to ask a scientific question, make a hypothesis and do the work to answer it and to have the answers increase our understanding of the natural world and potentially improve health and medicine was attractive to me.

**Patrice:** My hometown, in City Island, New York, sits alongside a remediated landfill. During my childhood, several children in the surrounding community were diagnosed with leukemia, prompting concerns about the role of environmental toxins in their illness. Driven by a desire to make a difference, I grappled with how best to support my community throughout my undergraduate studies. I resolved to pursue a career in experimental research, where I could actively investigate and test hypotheses, and contribute to our collective understanding of environmental challenges.

**How would you explain the main finding of your paper?**

**Abigail and Patrice:** Inorganic arsenic, a naturally occurring element found in bedrock, poses a significant health risk to populations exposed through unfiltered drinking water. Liver disease is one of the many health issues associated with increased exposure to inorganic arsenic. Despite numerous studies focusing on its immediate toxic effects, only a handful have investigated the long-term consequences. Our research paper demonstrates that developmental exposure to inorganic arsenic reduces the liver's ability to produce enzymes crucial for arsenic breakdown – a vital insight into the potential long-term health implications of arsenic exposure during early development.

Our research highlights the enduring effects of developmental exposure to inorganic arsenic.

**What are the potential implications of this finding for your field of research?**

**Abigail and Patrice:** Our research highlights the enduring effects of developmental exposure to inorganic arsenic. We found that zebrafish exposed to inorganic arsenic during development exhibited reduced mating success, a finding with significant implications for both human and animal populations residing in arsenic-endemic regions.

**Figure:**
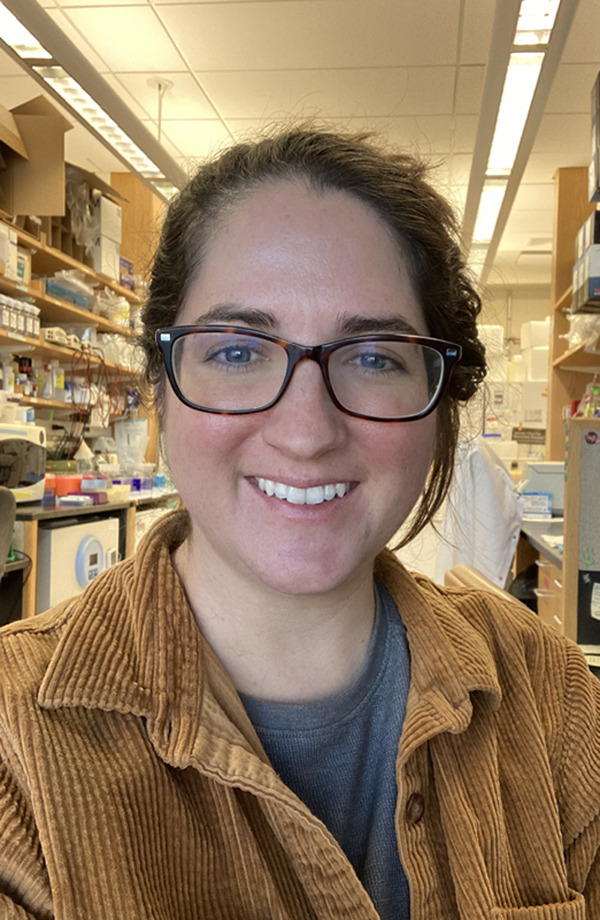
Patrice Delaney

**Which part of this research project was the most rewarding?**

**Abigail:** The most rewarding part for me was being mentored by incredibly hardworking and passionate scientists, learning to abandon a wrong hypothesis, and going where the science led.

**Patrice:** The project's most gratifying aspect was witnessing the independent intellectual contributions of my mentees, Abigail and Nouf Khan. Their ability to drive their own initiatives and infuse the project with their insights was rewarding. Through this experience, I've come to understand that while investing additional effort in experiments doesn't always yield better results, dedicating time to mentor talented undergraduates consistently leads to remarkable success.

**Figure:**
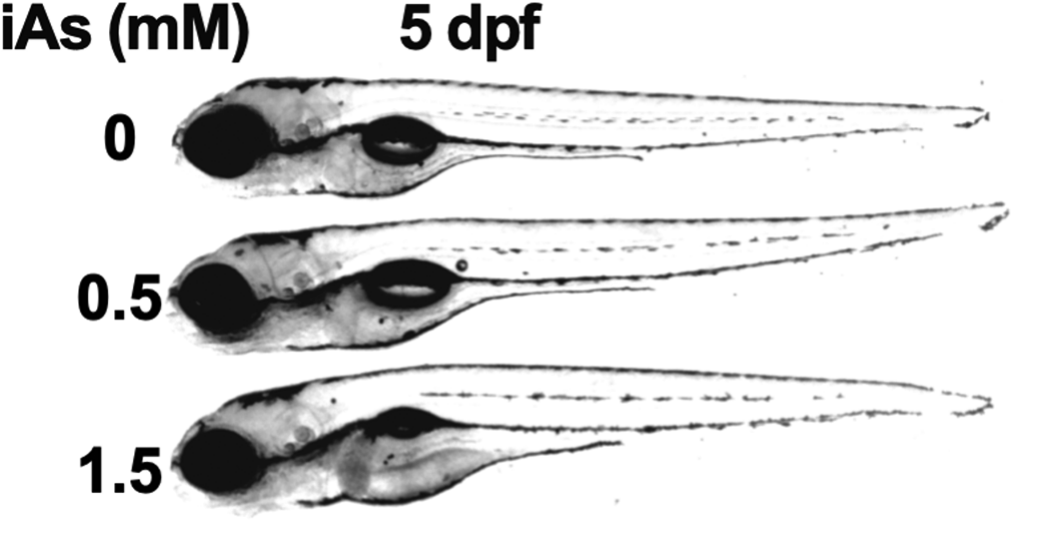
Zebrafish larvae at five days post-fertilization, following developmental exposure to inorganic arsenic concentrations of 0, 0.5, and 1.5 mM.

**What do you enjoy most about being an early career researcher?**

**Abigail:** I like that I'm surrounded by more experienced scientists who I learn from as they share their own unique journeys, mistakes, and successes. Also, I'm able to explore an array of research interests to better decide on what field of research that I'm most excited about.

**Patrice:** I appreciate the early-career stage for the freedom it offers to investigate high-risk, high-reward scientific questions. Moreover, I find reassurance in knowing that I have the support of a seasoned expert and team.

**What piece of advice would you give to the next generation of researchers?**

**Abigail:** Don't be afraid to ask questions, even the silly ones, and for opportunities.

**Patrice:** More than anything, during the early phase of your career, prioritize finding a mentor and a supportive team who possess both the expertise and empathy to guide you through the highs and lows of research.

**What's next for you?**

**Abigail:** I'm currently in my first year of graduate school at Yale, taking classes and doing lab rotations. I'm excited to choose a thesis mentor and lab, and to work on a research project for the next couple of years of my PhD.

**Patrice:** As of September 2023, I joined Dr Wolfram Goessling's lab at Harvard Medical School. Building on my training in the Sadler Lab, my goal is to investigate the contributions of anthropogenic contaminants in liver disease.
